# Sobolev’s embedding on time scales

**DOI:** 10.1186/s13660-018-1730-y

**Published:** 2018-06-19

**Authors:** Naveed Ahmad, Hira Ashraf Baig, Ghaus ur Rahman, M. Shoaib Saleem

**Affiliations:** 1 Institution of Business Administration, Karachi, Pakistan; 2 Department of Mathematics and Statistics, University of Swat, KPK, Pakistan; 3 Department of Mathematics, University of Education, Okara, Pakistan

**Keywords:** 34N05, 47N10, 46E35, 57R40, Sobolev spaces, Time scales, Embedding

## Abstract

For $1 \leq p < n $, the embeddings of Sobolev spaces $W^{1,p}_{\Delta }(\Omega_{\mathbb{T}^{n}})$ of functions defined on an open subset of an arbitrary time scale $\mathbb{T}^{n}$, $n\geq1$, endowed with the Lebesgue Δ-measure have been developed in (Agarwal et al. in Adv. Differ. Equ. 2006:38121, 2006) for $n=1$ and later generalized to arbitrary $n \geq1$ in (Su et al. in Dyn. Partial Differ. Equ. 12(3):241–263, 2015). In this article we present the embeddings of Sobolev spaces $W^{1,p}_{\Delta}(\Omega_{\mathbb {T}^{n}})$ for $n\leq p \leq\infty$ and then, using these embeddings, we develop general Sobolev’s embedding for the Sobolev spaces $W^{1,p}_{\Delta}(\Omega_{\mathbb{T}^{n}})$ on time scales, where *k* is a non-negative integer and $1\leq p \leq\infty$.

## Introduction

Before 1988 discrete and continuous analyses were independently treated as two different branches of mathematics. In order to combine differential as well as integral calculus with the notion of finite differences calculus, German mathematician Stefan Hilger introduced a theory. This new approach is known as time scale theory. It unites two approaches of dynamic modeling, difference and differential equations. In principle, these two approaches are special cases of a more general theory of time scale calculus. Time scale calculus is applicable in a field where dynamic processes can be described using discrete or continuous models. The applications of this theory are substantial and have received a lot of attention over the last few years. The most important among them is a system of dynamic equations. Moreover, it has various applications in biology, engineering, economics, physics, neutral network, and social sciences. For further details and basic notions of time scale calculus, we refer to [3–6].

Sobolev spaces are among the fundamental tools of functional analysis. They are used in variational methods to solve ordinary/partial differential equations or difference equations. In spite of this, the theory for functions defined on an arbitrary bounded interval of the real numbers has been established [7, 8]. Nevertheless, for functions defined on an arbitrary time scale, it appears that the study has not given too much attention. In [9] the authors studied the Sobolev spaces on time scales. Moreover, the authors presented some applications of their work by making use of variational method. To illustrate the feasibility and effectiveness of the existence results, some examples are provided at the end of the paper.

Motivated by the above work, in this paper we will study properties of Sobolev spaces. Also, general Sobolev’s embedding will be developed along with important embedding called Morrey’s inequality for the Sobolev spaces of functions defined on an open subset of an arbitrary time scale $\mathbb{T}^{n}$. These embeddings actually relate the Sobolev spaces with the space of Hölder continuous functions on an open domain of $\mathbb{R}^{n}$.

Beside the introductory section, the paper consists of three sections. In Sect. 2 we provide definitions of some basic notions related to an *n*-dimensional time scale. Also, some function spaces, like a space of Hölder continuous functions, $L_{p}$ spaces, Sobolev spaces, and some important results connected to *n*-dimensional time scale, are presented. Finally, in Sect. 3 we develop main results of the manuscript, the Morrey type inequality, and the general Sobolev’s embedding on an *n*-dimensional time scale.

## Preliminaries

Here, we recall some elementary results which will be used throughout the article.

Suppose *n* to be a positive integer. For each $i\in\lbrace1, 2,\ldots, n\rbrace$, let $\mathbb{T}_{i}$ denote a time scale, that is, a non-empty closed subset of $\mathbb{R}$. Set

$$\mathbb{T}^{n}= \mathbb{T}_{1} \times\mathbb{T}_{2} \times\cdots\times \mathbb{T}_{n} = \bigl\lbrace t=(t_{1}, t_{2},\ldots, t_{n}) : t_{i} \in \mathbb{T}_{i}, i=1,2,\ldots,n\bigr\rbrace , $$

we call $\mathbb{T}^{n}$ an *n*-dimensional time scale. The set $\mathbb {T}^{n}$ is a complete metric space with the metric *d* defined by

$$d(t,s)= \Biggl(\sum_{i=1}^{n} | t_{i} - s_{i}|^{2} \Biggr)^{1/2} \quad \mbox{for }t,s \in\mathbb{T}^{n}. $$

Denote by $\sigma_{i}$ and $\rho_{i}$ the forward and backward jump operators defined on $\mathbb{T}_{i}$. Specially, for $t_{i}\in \mathbb{T}_{i}$, the forward jump operator $\sigma_{i}:\mathbb{T}_{i}\longrightarrow\mathbb{T}_{i}$ is defined by

$$\sigma_{i}(t_{i})= \inf \lbrace s_{i} \in \mathbb{T}_{i}: s_{i} > t_{i} \rbrace; $$

and the back jump operator $\rho_{i}:\mathbb{T}_{i} \longrightarrow \mathbb{T}_{i}$ is defined by

$$\rho_{i}(t_{i})= \sup \lbrace t_{i} \in \mathbb{T}_{i}: s_{i} < t_{i} \rbrace. $$

In this definition we put $\sigma_{i}( \max \mathbb{T}_{i})= \max \mathbb{T}_{i}$ if $\mathbb{T}_{i}$ has a finite maximum and $\rho _{i}( \min \mathbb{T}_{i})= \min \mathbb{T}_{i}$ whenever $\mathbb {T}_{i}$ has finite minimum. Also, we call each $t_{i}\in\mathbb {T}_{i}$ the right-scattered element in $\mathbb{T}_{i}$ if $\sigma _{i}(t_{i}) > t_{i}$, right-dense element in $\mathbb{T}_{i}$ if $\sigma_{i}(t_{i}) = t_{i}$, where $t_{i} < \max \mathbb{T}_{i}$, left-scattered in $\mathbb{T}_{i}$ if $\rho_{i}(t_{i}) < t_{i} $, and left-dense in $\mathbb{T}_{i}$ if $\rho_{i}(t_{i}) = t_{i}$, where $t_{i} > \min \mathbb{T}_{i}$. If $\mathbb{T}_{i}$ has a left-scattered maximum *M*, then we define $\mathbb{T}_{i}^{k}= \mathbb{T}_{i}\setminus\lbrace M \rbrace$, otherwise $\mathbb{T}_{i}^{k}= \mathbb{T}_{i}$. When $\mathbb {T}_{i}$ has a right-scattered minimum *m*, then $(\mathbb {T}_{i})_{k}= \mathbb{T}_{i}\setminus\lbrace m \rbrace$, otherwise $(\mathbb{T}_{i})_{k}= \mathbb{T}_{i}$.

Assume a function $f:\mathbb{T}^{n} \longrightarrow\mathbb{R}$. The partial delta derivative of *f* with respect to $t_{i} \in(\mathbb {T}^{n})^{k}$ is defined as

$$\lim_{ s_{i} \longrightarrow t_{i} , s_{i} \neq\sigma_{i}(t_{i})} \frac{f( t_{1}, \ldots , t_{i-1} , \sigma_{i}(t_{i}) , t_{i+1} , \ldots , t_{n}) - f(t_{1}, \ldots , t_{i-1} , s_{i} , t_{i+1} , \ldots , t_{n})}{\sigma_{i}(t_{i}) - s_{i} } $$

whenever the limit exists, and denoted by $\frac{\partial f(t)}{\Delta _{i} t_{i}}$. Furthermore, the second order partial delta derivative of *f* is denoted as $\frac{\partial^{2} f(t)}{\Delta_{i} t_{i}^{2}}$ or $\frac{\partial^{2} f(t)}{\Delta_{i} t_{i} \Delta_{j} t_{j}}$. In the same fashion, one can define a higher order delta derivative.

In addition to the above, the partial nebla derivative of *f* with respect to the independent variable $t_{i} \in(\mathbb{T}^{n})_{k}$ is defined as

$$\lim_{ s_{i} \longrightarrow t_{i} , s_{i} \neq\rho_{i}(t_{i})} \frac{f( t_{1}, \ldots , t_{i-1} , \rho_{i}(t_{i}) , t_{i+1} , \ldots , t_{n}) - f(t_{1}, \ldots , t_{i-1} , s_{i} , t_{i+1} , \ldots , t_{n})}{\rho _{i}(t_{i}) - s_{i} } $$

provided the limit exists and is denoted by $\frac{\partial f(t)}{\rho _{i} t_{i}}$. The second order partial nebla derivative of *f* is denoted as $\frac{\partial^{2} f(t)}{\rho_{i} t_{i}^{2}}$ or $\frac {\partial^{2} f(t)}{\rho_{i} t_{i} \rho_{j} t_{j}}$. Higher order partial nebla derivatives are similarly defined. Combining both delta and nebla derivatives, we can define the mixed derivatives. For instance, a second order mixed derivative is denoted by $\frac {\partial^{2} f(t)}{\Delta_{i} t_{i} \rho_{j} t_{j}}$ or $\frac {\partial^{2} f(t)}{\rho_{i} t_{i} \Delta_{j} t_{j}}$. Hence, for any multi index $\alpha= (\alpha_{1},\ldots,\alpha_{n})$ of order $|\alpha| = \alpha_{1} + \cdots + \alpha_{n}$, we define

$$D^{\alpha}_{\Delta}f = \frac{\partial^{|\alpha|}f}{\Delta_{1} t_{1}^{\alpha_{1}} \cdots \Delta_{n} t_{n}^{\alpha_{n}}}. $$

If *k* is a non-negative integer, then

$$D^{k}_{\Delta}f = \bigl\lbrace D^{\alpha}_{\Delta}f : |\alpha|= k \bigr\rbrace , $$

i.e., the set of all delta partial derivatives of order *k* with

$$\bigl\vert D^{k}_{\Delta}f \bigr\vert = \biggl(\sum _{ \vert \alpha \vert =k} \bigl\vert D^{\alpha }_{\Delta}f \bigr\vert ^{2} \biggr)^{1/2}. $$

For $k=1$, the elements of $D_{\Delta}f$ can be seen in the form of a vector:

$$D_{\Delta}f = \biggl( \frac{\partial f}{\Delta t_{1}} ,\ldots, \frac {\partial f}{\Delta t_{n}} \biggr). $$

For a Δ-measurable set $\mathbb{E}_{\mathbb{T}} \subset \mathbb{T}^{n}$ and a Δ-measurable function $f:\mathbb {E}_{\mathbb{T}} \longrightarrow\mathbb{R}$, the corresponding Lebesgue Δ-integral of *f* over $\mathbb{E}_{\mathbb{T}}$ will be denoted by

$$\int_{\mathbb{E}_{\mathbb{T}}} f( t_{1} , t_{2},\ldots, t_{n} ) \Delta t_{1} \Delta t_{2} , \ldots , \Delta t_{n} \quad \mbox{or}\quad \int_{\mathbb{E}_{\mathbb {T}}} f(t) \Delta t \quad \mbox{or}\quad \int_{\mathbb{E}_{\mathbb{T}}} f(t) \mu _{\Delta}, $$

where $\mu_{\Delta}$ represents the Lebesgue measure [10].

### Some function spaces and results on time scales

Let $\Omega_{\mathbb{T}^{n}}$ be an open subset of $\mathbb{T}^{n} $, $n\geq1$.

$$\begin{aligned}& C^{k}( \Omega_{\mathbb{T}^{n}} ) = \lbrace u : \Omega_{\mathbb{T}^{n}} \longrightarrow\mathbb{R} : u \mbox{ is $k$-times continuously differentiable on } \Omega_{\mathbb{T}^{n}} \rbrace, \\& C^{\infty}( \Omega_{\mathbb{T}^{n}} ) = \lbrace u : \Omega _{\mathbb{T}^{n}} \longrightarrow\mathbb{R} : u \mbox{ is infinitely differentiable on } \Omega_{\mathbb{T}^{n}} \rbrace= \bigcap_{k=0}^{\infty} C^{k}( \Omega_{\mathbb{T}^{n}}). \end{aligned}$$

Moreover, we define some other auxiliary spaces

$$\begin{aligned}& C^{k}_{c}( \Omega_{\mathbb{T}^{n}} ) = \mbox{Functions in }C^{k}( \Omega_{\mathbb{T}^{n}} )\mbox{ with compact support}, \\& C^{\infty}_{c}( \Omega_{\mathbb{T}^{n}} ) = \mbox{Functions in }C^{\infty }( \Omega_{\mathbb{T}^{n}} )\mbox{ with compact support}, \\& \operatorname{BC}( \Omega_{\mathbb{T}^{n}} ) = \mbox{Space of all continuous and bounded functions on }\Omega_{\mathbb{T}^{n}}. \end{aligned}$$

For $u \in \operatorname{BC}( \Omega_{\mathbb{T}^{n}} ) $ and $0< \gamma\leq1$, let

$$\| u \|_{C( \Omega_{\mathbb{T}^{n}} )} := \sup_{t \in\Omega _{\mathbb{T}^{n}}} \bigl\vert u(t) \bigr\vert \quad \mbox{and}\quad [u]_{\gamma}:= \sup_{t , s \in\Omega _{\mathbb{T}^{n}},t \neq s} \biggl\lbrace \frac{| u(t)-u(s) |}{| t-s |^{\gamma}} \biggr\rbrace . $$

If $[u]_{\gamma}<\infty$, then *u* is Hölder continuous with Hölder exponent *γ*. The collection of *γ*-Hölder continuous functions on $\Omega_{\mathbb{T}^{n}}$ will be denoted by

$$C^{0,\gamma}(\Omega_{\mathbb{T}^{n}}):= \bigl\lbrace u \in \operatorname{BC}( \Omega _{\mathbb{T}^{n}}) : [u]_{\gamma}< \infty\bigr\rbrace , $$

and for $u \in C^{0,\gamma}(\Omega_{\mathbb{T}^{n}}) $ we can define the norm by

2.1 $$ \| u \|_{C^{0,\gamma}(\Omega_{\mathbb{T}^{n}})} := \| u \|_{C( \Omega_{\mathbb{T}^{n}} )} + [u]_{\gamma}. $$

For $\gamma=1$, the function *u* is said to be a Lipschitz continuous function.

#### Theorem 2.1

*The functions space* $C^{0,\gamma}(\Omega_{\mathbb{T}^{n}})$ *is a Banach space with respect to the norm defined in* (2.1).

#### Proof

For $u,v \in C^{0,\gamma}(\Omega_{\mathbb{T}^{n}})$,

$$\begin{aligned}{} [u+v]_{\gamma} =& \sup_{t,s \in\Omega_{\mathbb{T}^{n}} , t\neq s } \biggl\lbrace \frac{ | v(s)+u(s)-v(t)-u(t)|}{|t-s|^{\gamma}} \biggr\rbrace \\ \leq& \sup_{t,s \in\Omega_{\mathbb{T}^{n}} , t\neq s } \biggl\lbrace \frac{ | v(s)- v(t)|+|u(t)-u(s)|}{|t-s|^{\gamma}} \biggr\rbrace \\ \leq& [v]_{\gamma} + [u]_{\gamma} \end{aligned}$$

and for $\lambda\in\mathbb{R}$,

$$[\lambda u]_{\gamma} = \sup_{t , s \in\Omega_{\mathbb{T}^{n}},t \neq s} \biggl\lbrace \frac{| \lambda u(t)- \lambda u(s) |}{| t-s |^{\gamma}} \biggr\rbrace = |\lambda|[u]_{\gamma}. $$

This shows that $[\cdot]_{\gamma}$ is a semi-norm on $C^{0,\gamma }(\Omega_{\mathbb{T}^{n}})$, and therefore $\| u \|_{C^{0,\gamma }(\Omega_{\mathbb{T}^{n}})}$ defined in (2.1) is a norm. To see that $C^{0,\gamma}(\Omega_{\mathbb{T}^{n}})$ is complete, let $u_{m}$ be a Cauchy sequence in $C^{0,\gamma}(\Omega_{\mathbb {T}^{n}})$. Since $\operatorname{BC}(\overline{\Omega}_{\mathbb{T}^{n}})$ is complete, there exists $u \in \operatorname{BC}(\overline{\Omega}_{\mathbb{T}^{n}})$ such that $\| u-u_{m} \|_{C(\overline{\Omega}_{\mathbb {T}^{n}})}\rightarrow0$, $m \rightarrow\infty$.

For $t,s \in\Omega_{\mathbb{T}^{n}}$ with $t\neq s$,

$$\frac{| u(t)- u(s) |}{| t-s |^{\gamma}} = \lim_{m \rightarrow\infty }\frac{| u_{m}(t)- u_{m}(s) |}{| t-s |^{\gamma}} \leq \limsup _{m \rightarrow\infty} [u_{m}]_{\gamma} \leq \lim _{m \rightarrow \infty}\| u_{m} \|_{C^{0,\gamma}(\Omega_{\mathbb{T}^{n}})} < \infty, $$

and so we see that $u \in C^{0,\gamma}(\Omega_{\mathbb{T}^{n}})$. Similarly,

$$\begin{aligned} \frac{| u(t)- u_{m}(t)- ( u(s)- u_{m}(s)) |}{| t-s |^{\gamma}} =& \lim_{n \rightarrow\infty}\frac{| ( u_{n}- u_{m})(t)- ( u_{n}- u_{m})(s) |}{| t-s |^{\gamma}} \\ \leq& \limsup_{n \rightarrow\infty} [u_{n} - u_{m}]_{\gamma } \rightarrow0 ,\quad n\rightarrow\infty, \end{aligned}$$

showing $[u - u_{m}]_{\gamma}\rightarrow0$ as $n\rightarrow\infty$, and therefore $\lim_{n \rightarrow\infty} \| u - u_{m}\| _{C^{0,\gamma}(\Omega_{\mathbb{T}^{n}})}=0 $. □

The Hölder space $C^{k , \gamma}(\Omega_{\mathbb{T}^{n}})$ consists of those functions *u* that are *k* times continuously differentiable and whose *k*th-partial derivatives are Hölder continuous with exponent *γ*. The norm linear spaces, $C^{k , \gamma}(\Omega_{\mathbb{T}^{n}})$ are Banach spaces with the norm defined by

$$\| u \|_{C^{k , \gamma}(\Omega_{\mathbb{T}^{n}})} = \sup_{t \in \Omega_{\mathbb{T}^{n}}} \bigl|D^{\alpha}_{\Delta}u\bigr| + \sup_{t , s \in \Omega_{\mathbb{T}^{n}},t \neq s} \biggl\lbrace \frac{| u(t)-u(s) |}{| t-s |^{\gamma}} \biggr\rbrace . $$

#### Definition 2.2

([1])

For $p \in\mathbb{R}$ and $p\geq1$, the Lebesgue space $L_{\Delta}^{p}$ is defined as

$$ L_{\Delta}^{p}( \Omega_{\mathbb{T}^{n}} )= \biggl\lbrace u : \Omega _{\mathbb{T}^{n}}\longrightarrow\mathbb{R} : \int_{\Omega_{\mathbb {T}^{n}}} | u |^{p} \Delta t < + \infty \biggr\rbrace $$

equipped with the norm

$$ \| u \|_{L_{\Delta}^{p}(\Omega_{\mathbb{T}^{n}} )} = \biggl( \int _{\Omega_{\mathbb{T}^{n}}} | u |^{p} \Delta t \biggr)^{1/p}. $$

#### Remark 2.3

We know that the space $L_{\Delta}^{p}( \Omega_{\mathbb{T}^{n}} )$ is a Banach space with the norm defined above. Moreover, it is a Hilbert space for $p=2$, with the inner product defined by

$$\langle u , v \rangle_{L_{\Delta}^{2}( \Omega_{\mathbb{T}^{n}} )} = \int_{\Omega_{\mathbb{T}^{n}}} u(t)v(t) \Delta t. $$

#### Weak derivative

([1])

Suppose $u , v \in L^{1}_{\mathrm{loc} , \Delta} (\Omega_{\mathbb{T}^{n}})$ and *α* is multi-index. We say that *v* is *α*th-weak partial delta derivative of *u*, written as $D^{\alpha}_{\Delta}u$, provided

$$\int_{ \Omega_{\mathbb{T}^{n}}} u D^{\alpha}_{\Delta}\phi \Delta t = (-1)^{|\alpha|} \int_{ \Omega_{\mathbb{T}^{n}}} v \phi^{\sigma } \Delta t \quad \mbox{for all } \phi\in C_{c}^{|\alpha|} (\Omega_{\mathbb{T}^{n}}). $$

#### Sobolev spaces of order *k*

([1])

For $p\geqslant1$ and a non-negative integer *k*,

$$W^{k,p}_{\Delta}(\Omega_{\mathbb{T}^{n}}) = \bigl\lbrace u : \Omega_{\mathbb{T}^{n}}\longrightarrow\mathbb{R} : u^{\sigma} \in L^{p}_{\Delta}(\Omega_{\mathbb{T}^{n}})\mbox{ and }D^{\alpha}_{\Delta}u \in L^{p}_{\Delta}( \Omega_{\mathbb{T}^{n}}) , 0< |\alpha|\leqslant k \bigr\rbrace . $$

For $k=0$, $W^{k,p}_{\Delta}(\Omega_{\mathbb{T}^{n}})$ means $L_{\Delta}^{p}( \Omega_{\mathbb{T}^{n}} )$. It is obvious that $W^{k,p}_{\Delta}(\Omega_{\mathbb{T}^{n}})$ is a vector space. Sobolev spaces are Banach spaces under the norm defined by

$$\Vert u \Vert_{W^{k,p}_{\Delta}(\Omega_{\mathbb{T}^{n}})} = \biggl( \int_{\Omega_{\mathbb{T}^{n}}} \biggl( \bigl\vert u^{\sigma} \bigr\vert ^{p} + \sum_{0< |\alpha|\leqslant k} | D^{\alpha}_{\Delta}u |^{p} \biggr) \Delta t \biggr)^{1/p},\quad 1 \leqslant p < \infty. $$

We define the space $W^{k,p}_{0,\Delta}(\Omega_{\mathbb{T}^{n}})$ as the closure of $C^{\infty}_{c}(\mathbb{T}^{n})$ in $W^{k,p}_{\Delta }(\Omega_{\mathbb{T}^{n}})$ with the norm defined above, and it is also a Sobolev space of order *k*. For $p=2$, the spaces $W^{k,p}_{\Delta}(\Omega_{\mathbb{T}^{n}})$ are Hilbert spaces with the inner product

$$(u,v)_{W^{k,2}_{\Delta}(\Omega_{\mathbb{T}^{n}})} = \int_{\Omega _{\mathbb{T}^{n}}} \biggl( \bigl\vert u^{\sigma} \cdot v^{\sigma} \bigr\vert + \sum_{0< |\alpha|\leqslant k} D^{\alpha}_{\Delta}u \cdot D^{\alpha }_{\Delta}v \biggr) \Delta t. $$

We usually express $H^{k}_{\Delta} = W^{k,2}_{\Delta}(\Omega _{\mathbb{T}^{n}})$ and $H^{k}_{0,\Delta} = W^{k,2}_{0,\Delta }(\Omega_{\mathbb{T}^{n}})$.

#### Gagliardo–Nirenberg–Sobolev inequality

([2])

Assume $1 \leqslant p < \infty$. Then there is a constant *C* depending only on *p* and *n* such that

$$\Vert u \Vert_{L_{\Delta}^{p^{\ast}}( \mathbb{T}^{n})} \leq C \Vert D u \Vert_{L_{\Delta}^{p^{\ast}}( \mathbb{T}^{n})}\quad \mbox{for all }u \in C_{0}^{1}\bigl(\mathbb{T}^{n} \bigr), $$

where $p*= \frac{n p}{n-p}$ called the Sobolev conjugate of *p*.

#### Extension theorem

([2])

*Let* $\Omega_{\mathbb{T}^{n}} \subset \mathbb{T}^{n}$ *be an open bounded subset with* $\Omega_{\mathbb {T}^{n}} \subset\Omega_{\mathbb{T}^{n}}^{\prime}$ *and* $k \geqslant 1$. *If* $\partial\Omega_{\mathbb{T}^{n}} \in C^{k}$, *then any function* $u(t)\in W^{k,p}_{\Delta}(\Omega_{\mathbb{T}^{n}}) $ *has an extension* $u^{\prime}(t)\in\Omega_{\mathbb{T}^{n}}^{\prime}$ *with compact support*. *Moreover*,

$$\bigl\Vert u^{\prime} \bigr\Vert _{W^{k,p}_{\Delta}(\Omega^{\prime}_{\mathbb {T}^{n}})} \leqslant c_{2} \Vert u \Vert_{W^{k,p}_{\Delta}(\Omega _{\mathbb{T}^{n}})}, $$

*where the constant* $c_{2} > 0$ *does not depend on* *u*.

#### Average values

If $\Omega_{\mathbb{T}^{n}}$ is an open and bounded subset of $\mathbb{T}^{n}$, then the average value of the function *u* is defined as

$$\int_{\Omega_{\mathbb{T}^{n}}} u(s) \Delta s =\frac{1}{\mu_{\Delta } (\Omega_{\mathbb{T}^{n}} )} \int_{\Omega_{\mathbb {T}^{n}}} u(s) \Delta s = \mbox{Average of $u$ over the domain }\Omega_{\mathbb{T}^{n}} $$

and for a ball $B(t,r) \subset\Omega_{\mathbb{T}^{n}}$

$$\int_{B(t,r)} u(s) \Delta s =\frac{1}{\mu_{\Delta} ( B(t,r) )} \int_{B(t,r)} u(s) \Delta s = \mbox{Average of $u$ over the ball }B(t,r). $$

## Main results and discussions

This part of the paper is devoted to the main results obtained in the present manuscript. Assume that $n < p < \infty$. We will show that if $u \in W^{1,p}_{\Delta}(\Omega_{\mathbb{T}^{n}})$, then *u* is in fact Hölder continuous, after possibly re-defining on a set of measure zero. The time scale analogue of Morrey’s inequality is provided in the following theorem.

### Theorem 3.1

(Morrey’s inequality on time scales)

*Assume* $n < p \leq\infty$, *then there exists a constant* *C*, *depending only on* *p* *and* *n*, *such that*

3.1 $$ \| u \|_{C^{0,\gamma}(\mathbb{T}^{n})} \leq C \| u \| _{W^{1,p}_{\Delta}(\mathbb{T}^{n})} $$

*for all* $u\in C^{1}(\mathbb{T}^{n})$, *where* $\gamma\equiv1- n/p$.

### Proof

We prove this inequality in three steps.

*Step* 1. First choose any ball $B(t,r)\subset\mathbb{T}^{n} $. We claim there exists a constant *C*, depending only on *n*, such that

3.2 $$ \int_{B(t,r)}^{\mathrm{Avg}} \bigl\vert u(s)- u(t) \bigr\vert \Delta s \leq C \int_{B(t,r)}\frac {|Du(s)|}{|s-t|^{n-1}}\Delta s. $$

To prove this, fix any point $w \in\partial B(0,1) $. Then if $0<\eta<r$,

$$\begin{aligned} \bigl\vert u(t+\eta w) - u(t) \bigr\vert &= \biggl\vert \int_{0}^{\eta} \bigl( u(t+\xi w) \bigr)^{\Delta_{\xi}} \Delta\xi \biggr\vert \\ &= \biggl\vert \int_{0}^{\eta} Du(t+\xi w)\cdot w \Delta\xi \biggr\vert \\ &\leq \int_{0}^{\eta} \bigl\vert Du(t+\xi w) \bigr\vert \Delta\xi. \end{aligned}$$

Hence,

$$\begin{aligned} \int_{\partial B(0,1)} \bigl\vert u(t+\eta w) - u(t) \bigr\vert \Delta S &\leq \int _{0}^{\eta} \int_{\partial B(0,1)} \bigl\vert Du(t+\xi w) \bigr\vert \Delta S \Delta\xi \\ &= \int_{0}^{\eta} \int_{\partial B(0,1)} \bigl\vert Du(t+\xi w) \bigr\vert \frac{\xi ^{n-1}}{\xi^{n-1}} \Delta S \Delta\xi. \end{aligned}$$

Let $s=t+\xi w$, so that $\xi=|t-s|$. Then, converting the polar coordinates, we obtain

$$\begin{aligned} \int_{\partial B(0,1)} \bigl\vert u(t+\eta w) - u(t) \bigr\vert \Delta S &\leq \int _{B(t,\eta)} \frac{|Du(\eta)|}{|t-s|^{n-1}} \Delta s \\ &\leq \int_{B(t,r)} \frac{|Du(\eta)|}{|t-s|^{n-1}} \Delta s . \end{aligned}$$

Multiplying by $\eta^{n-1}$ and integrating from 0 to *r* with respect to *η*, we get

$$\int_{B(t,r)} \bigl\vert u(s) - u(t) \bigr\vert \Delta s \leq \frac{r^{n}}{n} \int _{B(t,r)} \frac{|Du(\eta)|}{|t-s|^{n-1}} \Delta s, $$

which asserts

$$ \int_{B(t,r)}^{\mathrm{Avg}} \bigl\vert u(s)- u(t) \bigr\vert \Delta s \leq C \int_{B(t,r)}\frac {|Du(s)|}{|s-t|^{n-1}}\Delta s. $$

*Step* 2. Fix $t\in\mathbb{T}^{n}$. Then, applying inequality (3.2) and Hölder’s inequality on time scales, we have

$$\begin{aligned} \begin{aligned} \bigl\vert u(t) \bigr\vert & \leq \int_{B(t,1)}^{\mathrm{Avg}} \bigl\vert u(t)- u(s) \bigr\vert \Delta s + \int _{B(t,1)}^{\mathrm{Avg}} \bigl\vert u(s) \bigr\vert \Delta s \\ & \leq C \int_{B(t,1)} \frac{ \vert Du(\eta) \vert }{ \vert t-s \vert ^{n-1}} \Delta s + C \Vert u \Vert_{L^{p}_{\Delta}(\mathbb{T}^{n})} \\ & \leq \biggl( \int_{\mathbb{T}^{n}} \vert Du \vert ^{p} \Delta s \biggr)^{1/p} \biggl( \int_{B(t,1)}\frac{ \Delta s}{ \vert t-s \vert ^{(n-1)\frac {p}{p-1}}} \biggr)^{\frac{p-1}{p}} + C \Vert u \Vert_{L^{p}_{\Delta }(\mathbb{T}^{n})} \\ & \leq \| u \|_{W^{1,p}_{\Delta}(\mathbb{T}^{n})}. \end{aligned} \end{aligned}$$

Thus,

3.3 $$ \bigl\vert u(t) \bigr\vert \leq\| u \|_{W^{1,p}_{\Delta}(\mathbb{T}^{n})}. $$

The last estimate holds since $p>n$ implies $(n-1)\frac{p}{p-1} < n $; therefore

$$\int_{B(t,1)} \frac{ \Delta s}{|t-s|^{(n-1)\frac{p}{p-1}}} < \infty. $$

As $t \in\mathbb{T}^{n}$ is arbitrary, inequality (3.3) implies

3.4 $$ \sup_{\mathbb{T}^{n}} |u| \leq C \Vert u \Vert_{W^{1,p}_{\Delta }(\mathbb{T}^{n})} . $$

*Step* 3. Next choose any two points $t,s\in\mathbb{T}^{n}$ and write $r:=|x-y|$.

Let $V:= B(t,r)\cap B(s,r)$. Then

3.5 $$ \bigl\vert u(t)-u(s) \bigr\vert \leq \int_{V}^{\mathrm{Avg}} \bigl\vert u(t)- u(z) \bigr\vert \Delta z + \int _{V}^{\mathrm{Avg}} \bigl\vert u(s)-u(z) \bigr\vert \Delta z. $$

But inequality (3.2) and Hölder’s inequality on time scales allow us to estimate

$$\begin{aligned} \int_{V}^{\mathrm{Avg}} \bigl\vert u(t)- u(z) \bigr\vert \Delta z & \leq C \int_{B(t,r)}^{\mathrm{Avg}} \bigl\vert u(t)- u(z) \bigr\vert \Delta z \\ & \leq \biggl( \int_{B(t,r)} \vert Du \vert ^{p} \Delta z \biggr)^{1/p} \biggl( \int_{B(t,r)}\frac{ \Delta z}{ \vert t-z \vert ^{(n-1)\frac{p}{p-1}}} \biggr)^{\frac{p-1}{p}} \\ & \leq C \bigl( r^{n-(n-1)\frac{p}{p-1}} \bigr)^{\frac{p-1}{p}} \Vert Du \Vert_{L^{p}_{\Delta}(\mathbb{T}^{n})} \\ & = C \Vert Du \Vert_{L^{p}_{\Delta}(\mathbb{T}^{n})}. \end{aligned}$$

Therefore,

3.6 $$ \int_{V}^{\mathrm{Avg}} \bigl\vert u(t)- u(z) \bigr\vert \Delta z \leq C r^{1 - \frac{n}{p}} \| Du \| _{L^{p}_{\Delta}(\mathbb{T}^{n})}. $$

Similarly, we can estimate

3.7 $$ \int_{V}^{\mathrm{Avg}} \bigl\vert u(s)- u(z) \bigr\vert \Delta z \leq C r^{1 - \frac{n}{p}} \| Du \| _{L^{p}_{\Delta}(\mathbb{T}^{n})}, $$

substituting (3.6) and (3.7) into (3.5) yields

$$\bigl\vert u(t)-u(s) \bigr\vert \leq C r^{1-\frac{n}{p}} \Vert Du \Vert_{L^{p}_{\Delta }(\mathbb{T}^{n})} = C | t-s |^{1-\frac{n}{p}} \Vert Du \Vert _{L^{p}_{\Delta}(\mathbb{T}^{n})}. $$

Thus,

$$\Vert u \Vert_{C^{0,1-\frac{n}{p}}(\mathbb{T}^{n})} = \sup_{t\neq s} \biggl\lbrace \frac{u(t)-u(s)}{|t-s|^{1-\frac{n}{p}}} \biggr\rbrace \leq C \Vert Du \Vert_{L^{p}_{\Delta}(\mathbb{T}^{n})}. $$

Using this inequality and (3.4), we get our desired estimate (3.1). □

### Remark 3.2

We say $u^{*}$ is a version of a given function *u* provided

$$u=u^{*}\quad \mbox{a.e. }\Omega_{T^{n}}. $$

### Theorem 3.3

*Let* $\Omega_{\mathbb{T}^{n}}$ *be a bounded*, *open subset of* $\mathbb {T}^{n}$, *and suppose* $\partial\Omega_{\mathbb{T}^{n}}$ *is* $C^{1}$. *Assume* $n< p\leq\infty$ *and* $u\in W^{1,p}_{\Delta}(\Omega_{\mathbb {T}^{n}})$. *Then* *u* *has a version* $u^{*}\in C^{0,\gamma}(\overline{\Omega}_{\mathbb{T}^{n}})$, *for* $\gamma=1-\frac{n}{p}$, *with the estimate*

$$\bigl\Vert u^{*} \bigr\Vert _{C^{0,\gamma}(\overline{\Omega}_{\mathbb{T}^{n}})} \leq \| u \|_{W^{1,p}_{\Delta}(\Omega_{\mathbb{T}^{n}})} . $$

*The constant* *C* *depends only on* *p*, *n*, *and* $\Omega_{\mathbb{T}^{n}}$.

### Proof

Since $\partial\Omega_{\mathbb{T}^{n}}$ is $C^{1}$, then for any $u \in W^{1,p}_{\Delta}(\Omega_{\mathbb{T}^{n}})$ there exists an extension $\overline{u} \in W^{1,p}_{\Delta}(\mathbb {T}^{n})$ such that

3.8 $$ \textstyle\begin{cases} \overline{u}= u in \Omega_{\mathbb{T}^{n}},\quad \overline{u}\mbox{ has compact support}, \\ \| \overline{u} \|_{W^{1,p}_{\Delta}(\Omega_{\mathbb{T}^{n}})} \leq C_{2} \| u \|_{W^{1,p}_{\Delta}(\Omega_{\mathbb{T}^{n}})}. \end{cases} $$

Since *u̅* has compact support, there exists a sequence of functions $u_{m}\in C^{\infty}_{c}(\mathbb{T}^{n})$ such that

3.9 $$ u_{m} \longrightarrow\overline{u} \quad \mbox{in }W^{1,p}_{\Delta}\bigl(\mathbb {T}^{n}\bigr) . $$

Now, according to Theorem 3.1, we have

$$\| u_{m} - u_{l} \|_{C^{0,1-\frac{n}{p}}(\mathbb{T}^{n})} \leq C \| u_{m} - u_{l} \| _{W^{1,p}_{\Delta}(\mathbb{T}^{n})} $$

for all $l, m \geq1$; whence there exists a function $u^{*} \in C^{0,1-\frac{n}{p}}(\mathbb{T}^{n})$ such that

3.10 $$ u_{m} \longrightarrow u^{*}\quad \mbox{in }C^{0,1-\frac{n}{p}}\bigl(\mathbb{T}^{n}\bigr) . $$

Incorporating (3.9) and (3.10), we see that $u^{*}=u$ a.e. on $\Omega_{\mathbb{T}^{n}}$; so that $u^{*}$ is a version of *u*. Theorem 3.1 also implies

$$\| u_{m} \|_{C^{0,1-\frac{n}{p}}(\mathbb{T}^{n})} \leq C \| u_{m} \| _{W^{1,p}_{\Delta}(\mathbb{T}^{n})}. $$

Assertions (3.9) and (3.10) yield

$$\bigl\Vert u^{*} \bigr\Vert _{C^{0,1-\frac{n}{p}}(\mathbb{T}^{n})} \leq C \| \overline{u} \| _{W^{1,p}_{\Delta}(\mathbb{T}^{n})}. $$

This inequality and (3.8) yield the complete proof. □

Now we are capable of developing the general Sobolev type inequalities on time scales.

### Theorem 3.4

(General Sobolev’s embedding on time scales)

*Let* $\Omega_{\mathbb{T}^{n}}$ *be a bounded open subset of* $\mathbb {T}^{n}$, *with a* $C^{1}$ *boundary*. *Assume* $u \in W^{k,p}_{\Delta }(\mathbb{T}^{n})$.

(i) *If* $k < \frac{n}{p} $, *then* $u \in L^{q}_{\Delta}(\Omega _{\mathbb{T}^{n}})$, *where* $\frac{1}{q} = \frac{1}{p} - \frac {k}{n}$ *and*
  3.11 $$ \| u \|_{L^{q}_{\Delta}(\Omega_{\mathbb{T}^{n}})} \leq C \| u \| _{W^{k,p}_{\Delta}(\Omega_{\mathbb{T}^{n}})} $$
  *the constant* *C* *depends only on* *k*, *p*, *n*, *and* $\Omega_{\mathbb {T}^{n}} $.
(ii) *If* $k > \frac{n}{p} $, *then* $u \in C^{k-[\frac{n}{p}]-1 ,\gamma }( \overline{\Omega}_{\mathbb{T}^{n}})$, *where*
  $$ \gamma= \textstyle\begin{cases} [\frac{n}{p} ] + 1 - \frac{n}{p}& \textit{if } [\frac{n}{p} ]\textit{ is not an integer}, \\ \textit{any positive number} < 1 &\textit{if } [\frac{n}{p} ]\textit{ is an integer} \end{cases} $$
  *and*
  3.12 $$ \| u \|_{C^{k-[\frac{n}{p}]-1 ,\gamma}( \overline{\Omega}_{\mathbb {T}^{n}})} \leq C \| u \| _{W^{k,p}_{\Delta}(\Omega_{\mathbb {T}^{n}})}, $$
  *the constant* *C* *depending only on* *k*, *p*, *n*, *γ*, *and* $\Omega _{\mathbb{T}^{n}} $.

### Proof

(i) Assume $k < \frac{n}{p}$. Moreover, if $D^{\alpha}u \in L^{p}_{\Delta}(\Omega_{\mathbb{T}^{n}})$ for all $|\alpha|=k$, then the Sobolev–Nirenberg–Gagliardo inequality on time scales implies that

$$ \bigl\| D^{\beta}u\bigr\| _{L^{p^{*}}_{\Delta}(\Omega_{\mathbb{T}^{n}})} \leq C \| u \| _{W^{k,p}_{\Delta}(\Omega_{\mathbb{T}^{n}})}\quad \mbox{whenever }|\beta |= k-1, $$

thus we have, $u \in W^{k-1,p^{*}}_{\Delta}(\Omega_{\mathbb {T}^{n}})$. Similarly, we find that $u \in W^{k-2,p^{**}}_{\Delta }(\Omega_{\mathbb{T}^{n}})$, where $\frac{1}{p^{**}}=\frac {1}{p^{*}}-\frac{1}{n} = \frac{1}{p} -\frac{2}{n}$. Continuing in this way, after *k* steps we eventually come to the point that $u \in W^{0,q}_{\Delta}(\Omega_{\mathbb{T}^{n}}) = L^{q}_{\Delta}(\Omega _{\mathbb{T}^{n}})$ for $\frac{1}{q} = \frac{1}{p} - \frac{k}{n}$. The required estimate (3.11) follows by multiplying the relevant estimates at each stage of the above argument.

(ii) Now assume that $k > \frac{n}{p}$ and $\frac{n}{p}$ are not integers. Then, as above, we see

3.13 $$ u \in W^{k-l,r}_{\Delta}(\Omega_{\mathbb{T}^{n}}) $$

for

3.14 $$ \frac{1}{r} = \frac{1}{p} -\frac{l}{n} $$

with $l< p < n$. We choose the integer *l* so that

3.15 $$ l < \frac{n}{p} < l+1, $$

that is, $l= [\frac{n}{p} ]$. Consequently, (3.14) and (3.15) imply $r=\frac{pn}{n-pl}>n$. Hence (3.13) and Morrey’s inequality on time scales imply that $D^{\alpha }u \in C^{0,1-\frac{n}{r}}( \overline{\Omega}_{\mathbb{T}^{n}})$ for all $|\alpha|\leq k -l-1$. Observe that $1-\frac{n}{r}=1-\frac {n}{p}+l= [\frac{n}{p} ] + 1-\frac{n}{p}$. Thus $u \in C^{k- [\frac{n}{p}]-1, [\frac{n}{p}]+1-\frac{n}{p}} (\overline{\Omega}_{\mathbb{T}^{n}})$, and the stated estimate follows easily.

Finally, suppose that $k > \frac{n}{p}$ and $\frac{n}{p}$ are integers. Set $l= [\frac{n}{p} ]-l = \frac{n}{p} - 1$. Consequently, we have, as above, $u \in W^{k-l,r}_{\Delta}(\Omega _{\mathbb{T}^{n}})$ for $r=\frac{pn}{n-pl}=n$. Hence the Sobolev–Nirenberg–Gagliardo inequality on time scales shows $D^{\alpha}u \in L^{q}(\Omega_{\mathbb{T}^{n}})$ for all $n\leq q <\infty$ and all $|\alpha|\leq k-l-1=k- [\frac{n}{p} ]$. Therefore, Morrey’s inequality on time scales further implies $D^{\alpha}u \in C^{0,1-\frac{n}{r}}( \overline{\Omega}_{\mathbb {T}^{n}})$ for all $n \leq q < \infty$ and all $|\alpha|\leq k - [\frac{n}{p} ]-1$. Consequently, $u \in C^{k-[\frac{n}{p}]-1 ,\gamma}( \overline{\Omega}_{\mathbb{T}^{n}})$ for $0 < \gamma< 1$. □

## Conclusion

We studied embedding for functions in Sobolev spaces $W^{1,p}_{\Delta }(\mathbb{T}^{n})$ for $n\leq p \leq\infty$ and after that developed general Sobolev’s embedding on an arbitrary time scale. In case of $\mathbb{T}=R$, the results coincide with the classical results on Sobolev spaces, see [11–13]. These embeddings are important for further discussion of Sobolev spaces on time scales.
